# Echoes of the Month

**Published:** 1924-07

**Authors:** 


### ECHOES OF THE MONTH.

### TESTS OF MENTAL EFFICIENCY.

It will probably come as a surprise to many to read some of the tests set to the children in Council Schools. Those failing to pass them are, we understand, considered to be mentally deficient. A child of four has to discriminate between a circle, a square, a triangle and other mathematical forms. He or she must also draw a square correctly, repeat sentences of twelve or thirteen syllables, and inform the teacher:—" What you must do: When you are sleepy; When you are cold; When you are hungry." A child of seven has to tie a bow-knot; to state the difference between fly and butterfly; stone and egg; wood and glass. It has also to copy the figure of a diamond. At eight he is set to disentangle the following problem:—" Let us suppose that your cricket ball has been lost in this round field. You have no idea what part of the field it is in. You don't know what direction it came from, how it got there, or with what force it came. All you know is that the ball is lost somewhere in the field. Now take this pencil and mark out a path to show me how you would come to the ball so as to be sure not to miss it. Begin at the gate and show me what path you could take." We fear that the solutions offered would consign most children to the ranks of the mentally deficient.

### SECONDARY SCHOOL BUILDINGS DOOMED.

Speaking at the opening of the Barr Hill Open Air School, Salford, Dr. Osborne said that the type of present secondary school building was doomed; that what was good for a tuberculous child was good also for a healthy child. He, too, must shortly have open-air schools and work in free air and sunlight unimpeded by glass or, indeed, any obstacle whatever. Windows did much harm in excluding beneficial rays of light, and why should they withhold from the ordinary child conditions which benefited the delicate child so much? He firmly believed that in ordinary elementary schools we are evolving the condition of ill-health which filled the open-air schools.

### "FOR EVERY CHILD A CHANCE."

In his Shaftesbury Lecture Mr. J. L. Garvin said:—" 'For every child a chance' is the thought which, if rightly applied, offers one sure key to a better future for our own country and the world, perhaps the only key. When we sum up the relative conditions of the people in the time of the two great Exhibitions of 1851 and 1924, the difference seems like the whole change from deep night to broad day. Yet in this island it is estimated that we have no fewer than a million persons mentally and physically defective. We have another larger number vitiated by drunkenness. A still higher proportion of the population in this country, in this centre of the widest Empire that has ever existed, and in this year of its greatest Exhibition, is far below that standard of physique and that standard of training for the tasks of life which should belong to British citizens. Every boy and girl, whether they are to stay in Great Britain or elect to go to the Dominions, should have, in my conviction, some definite sense of the possibility of choice between a town life and a country life."

### MEDICAL BOOKS IN PUBLIC LIBRARIES.

Dr. Courtenay Dunn, of Torquay, is anxious that medical books should be banned from public libraries and has asked the authorities to remove books of this kind from their shelves. He points out that a certain class of people devour these books greedily, with disastrous results to themselves, and quotes two specific cases, female patients of his. One of these, he says, a neurotic, frequented a library a few weeks ago for the purpose of reading a book on medicine, with the consequence that she is now an inmate of an asylum. Mr. R. H. Halliday, the librarian, agrees with Dr. Courtenay Dunn.

### LIVINGSTONE COLLEGE.

At Commemoration Day at Livingstone College the chair was taken by Sir William J. Simpson, C.M.G., the well-known authority on Tropical Medicine and Hygiene. The Treasurer said that the financial side of the work of Livingstone College for the last few years had been one of considerable anxiety. He referred to the fact that the College has to depend on the fees of students and the subscriptions of a few friends for its maintenance. It is necessary to increase the number of students, and until that is an accomplished fact further subscriptions are called for. The Chairman said that the course at Livingstone College is an excellent one, and one that will be most useful to the missionary in order to teach him how to care for his own health, and to get into the hearts of the people amongst whom he works. Dr. Livingstone, in whose honour the College was founded, was not only a great missionary and explorer, who sacrificed his life for his work, but he was also a keen observer of nature. He was not the first to note the ravages of the tsetse fly on cattle and horses, but owing to his medical training he was able to bring this to the knowledge of the public, and gave an excellent description of the tsetse fly.

### THE SEARCH AFTER A CANCER CURE.

An article has been published in the Philadelphia "North American"—it has been withheld for fifteen months "in the interests of science"—in which it is claimed that the cause of cancer has been discovered and that the treatment, while still in the experimental stages, is "producing remarkable results." The discoverer, Dr. T. J. Glover, a Canadian, it is stated, has established scientifically that the disease is due to a micro-organism, and he has succeeded in isolating the germ and developing an antitoxic serum. During the past two years, it is claimed, the serum has been tested in 200 cases of cancer in its commonest and most destructive form, and in the majority favourable results were secured. In some instances the subjects were discharged as cured.

### NO MORE UNDERGROUND OFFICES.

The abolition of the use of underground rooms as offices after January 1, 1928, is one of the proposals of the Offices Regulation Bill. No underground room shall be used as an office unless it was so used when the Bill was passed. The main clause provides that every office must be kept in a cleanly state, free from effluvia, adequately lighted, and provided with an adequate supply of pure drinking water. The floors shall be cleaned thoroughly once at least within every seven days, and the windows within every twenty-eight days. The times for limewashing or papering are also laid down. An office will, under the Bill, be deemed to be so overcrowded as to be dangerous or injurious to the health of the persons employed therein if the number of cubic feet of space in any room bears to the number of persons employed at one time in the room a proportion less than 600 to every person.

### THE TRIUMPH OF RESEARCH.

Addressing a meeting of the Middlesex Hospital Medical Society which has recently celebrated its 150th anniversary, Sir James Kingston Fowler said that he thought it a great privilege to have been able to watch the steady progress made in medical science during the last 50 years. He believed the period would prove as great an epoch in the history of medicine as was the Elizabethan in the history of literature. Many epidemics formerly common were now unknown, and in 1923 no case of enteric was brought to the hospital. "Think," he said, "of the Boer War and the Bloemfontein camp and contrast it with the result in the late war of anti-typhoid inoculation." Sir James remarked that if one word could recall the greatest triumph of medical science since the Society entered its centenary that word was "research."

### INFANT MORTALITY AT ITS HIGHEST.

Dr. Feldman, writing in "National Health" on the development of the Infant Welfare movement, says that "the first asylum for abandoned infants was founded by Archbishop Datheus in 787 A.D. Between the 11th and 15th centuries many foundling hospitals were established in different parts of the world. In 1523 the Hôtel Dieu of Lyons, the oldest hospital in France, began to take in children. A prominent pioneer in the 17th century was St. Vincent de Paul who, in 1639, put on a firm foundation the asylum for exposed infants, started by an unknown woman of Paris. The asylum was endowed in 1670 by Louis XIV. In England, infantile mortality was very high in the 17th and 18th centuries, but the mortality amongst breast-fed babies was only a third of that amongst infants artificially fed. In the Dublin Foundling Hospital only 45 infants survived out of 10,272 admitted during the 21 years 1775–1796, giving a mortality of no less than 99.6 per cent.!"

